# The combination of lactate level, lactate clearance and APACHE II score better predicts short-term outcomes in critically Ill patients: a retrospective cohort study

**DOI:** 10.1186/s12871-022-01878-0

**Published:** 2022-12-09

**Authors:** Yongmei Cao, Sijia Yao, Jiawei Shang, Feng Ping, Qin Tan, Zijun Tian, Weifeng Huang, Yingchuan Li

**Affiliations:** 1 Department of Critical Care Medicine, School of Medicine, Shanghai Tenth People’s Hospital, Tongji University, Shanghai, China; 2 Department of Anesthesiology, Shanghai Jiao Tong University Affiliated Sixth People’s Hospital, Xuhui District, No. 600, Yishan Road, Shanghai, 200233 China; 3 Department of Critical Care Medicine, Shanghai Jiao Tong University Affiliated Sixth People’s Hospital, Xuhui District, No. 600, Yishan Road, Shanghai, 200233 China

**Keywords:** Lactate, Lactate clearance, APACHE II score, Critically ill patients, Outcome

## Abstract

**Background:**

The mortality rate is high in critically ill patients due to the difficulty of diagnosis and treatment. Thus, it is very important to explore the predictive value of different indicators related to prognosis in critically ill patients.

**Methods:**

This was a retrospective cohort study of patients in the intensive care unit (ICU) of the Sixth People’s Hospital in Shanghai, China. A total of 1465 ICU patients had lactate values > 2.1 mmol/L at least once within 24 h of ICU admission, and arterial blood gas was monitored more than twice during the ICU stay.

**Results:**

The predictive value of lactate clearance at 24 h was not high, and the sensitivity and specificity were lower. The predictive value of the lactate level at baseline and the APACHE II score was higher than that of lactate clearance at 24 h in critically ill patients. The predictive value of the lactate level at baseline combined with the APACHE II score was higher than that of the lactate level at baseline or the APACHE II score alone. In addition, the predictive value of lactate clearance at 24 h combined with the APACHE II score was also significantly higher than that of lactate clearance at 24 h or the APACHE II score alone. In particular, the area under the ROC curve reached 0.900, the predictive value was markedly higher than that of the ROC alone, and the sensitivity and specificity were better when these three indicators were combined.

**Conclusions:**

The combination of lactate level, lactate clearance and APACHE II score better predicts short-term outcomes in critically ill patients.

## Introduction

The mortality rate of critically ill patients is high due to the complexity and seriousness of their conditions. Risk stratification is conducive to the timely prediction of predict the prognosis of patients [1, 2]. Therefore, it is of great clinical significance to explore some indicators to predict short and long-term outcomes and guide the treatment of critically ill patients.

Hypoxia is very common in critically ill patients. Hypoxia accelerates the progression of disease and leads to poor prognosis of patients. When combining max lactate at T24 with APACHE III, the lactate level is more sensitive and easier to detect and acts as an indicator to predict the prognosis of critically ill patients [3]. The imbalance between oxygen delivery and consumption will cause the accumulation of lactate, and lactate levels will rise sharply [4, 5]. In addition, hypoperfusion can also result in an increased lactate level. Therefore, lactate levels can always reflect the state of oxygen metabolism, and hyperlactatemia is usually caused by hypoxia and hypoperfusion [6, 7]. Moreover, the decrease of in lactate clearance also plays a key role in the process of hyperlactatemia, particularly in elderly individuals with liver insufficiency or acute liver disease [8–10]. However, in recent experimental studies, researchers found that lactate clearance is reduced significantly in patients with sepsis, even if hemodynamics remain steady and liver function is normal, which may be related to the decreased activity of pyruvate dehydrogenase [11]. Increased lactate production or decreased clearance will eventually lead to an increase in lactate levels, resulting in hyperlactatemia [12].

Importantly, a high lactate level is closely related to mortality risk. Some researchers suggest that when the lactate level is high, the patient’s prognosis is poor [13–15], and there is a dose-dependent relationship between the lactate level and the risk of mortality [16, 17]. In addition, there is a significant correlation between persistently high lactate levels and an increased risk of mortality [18–20]. Patients with higher baseline lactate levels have a higher risk of mortality, and thus, the initial lactate concentration better predicts the risk of patient mortality [21, 22]. In a large retrospective study by Bou et al., lactate level was an independent predictor of hospital mortality in critically ill patients, and higher lactate values were associated with a higher risk of hospital mortality and a longer hospital stay [23]. In previous studies, researchers have also investigated the prediction of blood lactate values in critically ill patients using the MIMIC-III and eICU-CRD datasets on the MIMIC-III cohort in a retrospective multicenter cohort study. The results show that elevations in initially obtained serum lactate levels are strong predictors of mortality in critically ill patients and identifying patients whose serum lactate levels are more likely to increase can alert physicians to improve care and guide them in determining the frequency of conducting blood tests [24]. Therefore, lactate levels are considered a valuable indicator of patient mortality and can be used to guide treatment [25–27].

However, there are few studies on the relationship between dynamic changes in lactate levels and risk of mortality in critically ill patients. A retrospective study showed that lactate dynamics at 6 h and time-weighted average lactate may predict survival beyond 30 days in critically ill patients [28]. The clinical utility of delta lactate was investigated to predict early in-hospital mortality in adult patients in a prospective, multicentric, cohort study, and it was observed that the assessment of the dynamic changes in lactate levels can be a quick and easy tool for determining the initial state and the short-term prognosis of a critical patient. Their results also showed that both a low lactate level and a lactate clearance of more than 10% were related to an increase in survival [29]. Hyperlactatemia, a dynamic indicator, has significant independent predictive value, and a higher average lactate level and continuous increases in lactate concentration during a 24-h period were related to higher mortality in patients. Dynamic monitoring of lactate levels is needed, preferably at intervals of 2 or 6 h or longer, but corresponding results have not yet been reliably confirmed [30].

Therefore, we conducted a retrospective analysis to explore and analyze the correlation of the dynamic lactate level and other indicators with the prognosis of critically ill patients. Through this study, we can provide evidence-based, valuable and effective indicators for clinical application.

## Materials and methods

### The aim, design and setting of the study

This was a retrospective cohort study to explore the predictive value of different indicators related to prognosis in critically ill patients. The data were obtained from the Department of Critical Care Medicine of the Sixth People’s Hospital in Shanghai from Dec 2018 to May 2019. The study was approved by the Ethics Committee of Shanghai Sixth People’s Hospital Affiliated to Shanghai Jiao Tong University, Shanghai, China (No. 2019-KY-005(K)) and was registered in the Chinese Clinical Trial Registry (ChiCTR1800020252). The data were anonymous and the requirement for informed consent was therefore waived.

### Inclusion and exclusion criteria

Data were collected from critically ill patients who stayed in the intensive care unit for more than 24 h and whose lactate levels were abnormal. Patients were included in the study if they were treated in the ICU for more than 24 h, underwent arterial blood gas monitoring more than twice, and had abnormal lactate levels in their arterial blood (> 2.1 mmol/L) at least once within 24 h of ICU admission. We excluded patients with whose medical charts revealed implausible or incomplete data, those whose arterial blood gas analysis was conducted less than twice within 24 h, those with normal lactate levels, those who stayed in the ICU for less than 24 h, and those who receiving only conservative treatment but not invasive treatment strategy. For all enrolled patients, we collected the following clinical data: general characteristics, such as name, sex, and age; length of hospital stay (days); length of ICU stay (days); survival or death during hospitalization; diagnosis; type of operation; original department at admission; discharging department; baseline (0-h) and 24-h blood gas analysis results in the ICU; preoperative and postoperative liver function; preoperative and postoperative renal function; APACHE II score; mechanical ventilation; 24-h lactate clearance; reasons for admission (Elective surgery, emergency surgery or urgent events, such as sepsis, shock, post cardiopulmonary resuscitation, respiratory failure, acute myocardial infarction); Analgesia/sedation; diagnosis and the terms of operation and co-morbidities.

### Mortality assessment

The overall ICU mortality for all patients with lactate > 2.1 mmol/L within 24 h and for all subcategories was assessed. The survival rate of the patients during hospitalization, including survival or death in the hospital during or after ICU admission, was assessed.

### Lactate clearance (LC)

Lactate clearance (percent) was defined using the following formula: the lactate level at baseline presentation (hour 0) minus the lactate level at hour 24, divided by the lactate level at baseline and multiplied by 100. A positive value denotes a decrease or clearance of lactate, whereas a negative value denotes an increase in the lactate level after 24 h.

$${\mathrm{Lactate clearance}}^{24\mathrm{hrs}}={(\mathrm{Lactate}}^{\mathrm{hour }0}-{\mathrm{Lactate}}^{\mathrm{hour }24})\times 100/{\mathrm{Lactate}}^{\mathrm{hour }0}$$

### Statistical analysis

The statistical analysis of the data was performed using SPSS 20.0 software. The Kolmogorov–Smirnov test or Kruskal–Wallis rank sum test was used for quantitative data. Classification variables were expressed in the form of N (%). A chi-square test was used to compare the differences between the two groups, and binary logistic regression analysis was used to assess risk factors that might be related to the outcome of critically ill patients. All models were calculated and analyzed using the Hosmer–Lemeshow test and a correction for multiple comparisons was performed. The value of multiple indicators for predicting the outcomes in critically ill patients was assessed by calculating the area under the receiver operating characteristic curve (AUC), and the threshold was determined by calculating the maximum Youden’s index for sensitivity and specificity. A two-tailed *P* value < 0.05 was considered statistically significant.

## Results

### Participants and characteristics

**Fig. 1:**
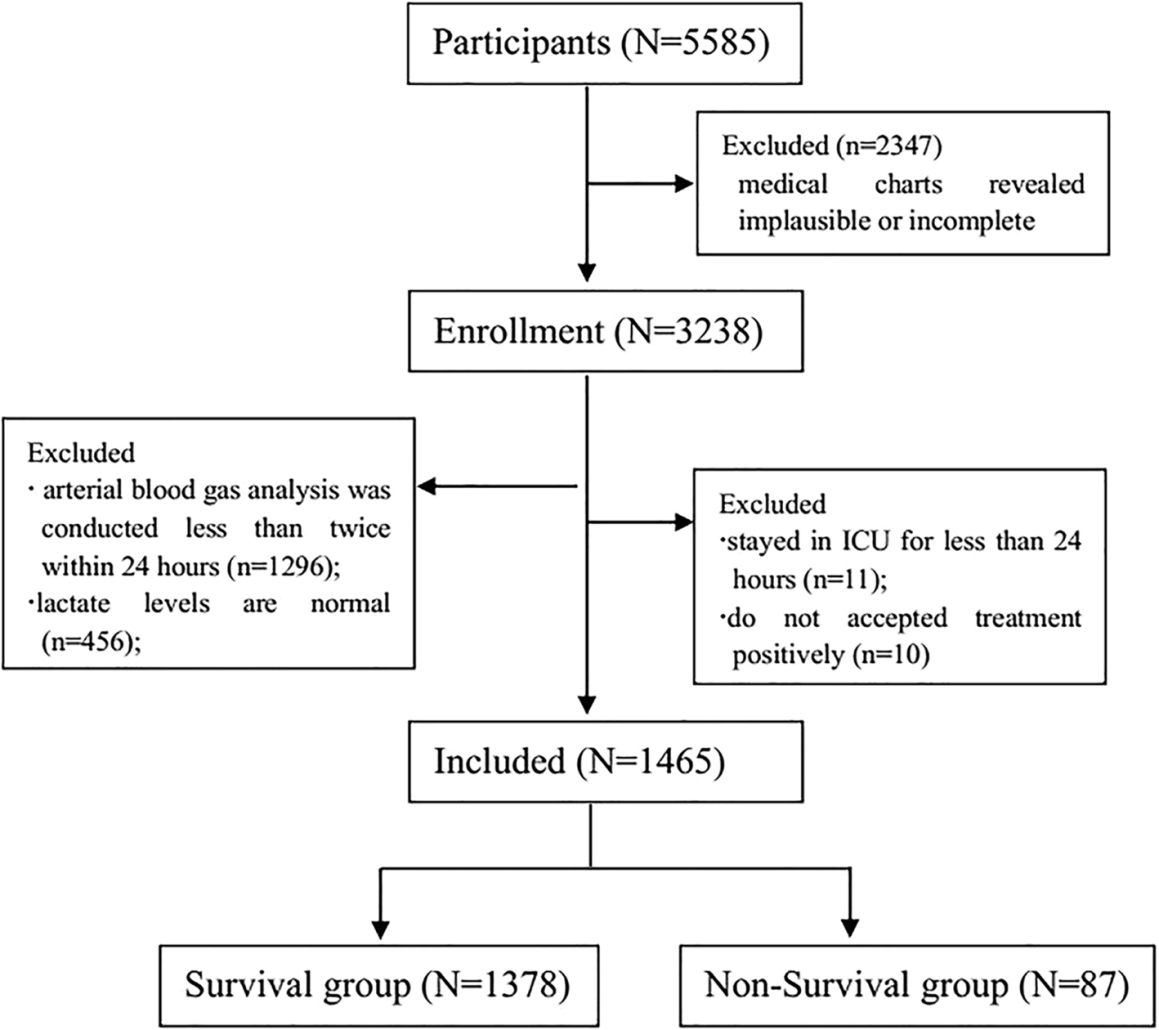
Flow of Participants Through the Study. A total data of 5585 participants, were collected from the HIS database. A total of 2344 patients whose medical charts revealed implausible or incomplete and 1773 patients who met the exclusion criteria were excluded. Finally, 1465 patients were included in the clinical trial

Data for 5585 participants who stayed at the Department of Critical Care Medicine at the Sixth People’s Hospital in Shanghai were collected from the Health Information System (HIS) database. A total of 2347 patients whose medical charts revealed implausible or incomplete data and 1773 patients who met the exclusion criteria were excluded. Ultimately, 1465 patients were included in the clinical trial (Fig. 1). According to outcome (survival or death), the patients were divided into two groups (the survival group and the non-survival group); patient characteristics are shown in Table 1. The mean age was older in the non-survival group and the proportion of males, emergency surgery and urgent events admitted to ICU was more in the non-survival group than in the survival group. The ICU length of stay, hospital length of stay, APACHE II score, and lactate level at baseline and 24 h after patients were admitted to the ICU were significantly higher in the non-survival group than in the survival group, and the lactate clearance at 24 h was lower in the non-survival group than in the survival group. The proportion of patients with mechanical ventilation in the survival group was higher than that in the non-survivor group, which may be due to the higher proportion of patients with elective surgery in the survival group, while the higher proportion of patients undergoing emergency surgery or urgent events in the non-survivor group. Both before and after surgery, the proportion of patients with abnormal liver (An abnormality of glutamic pyruvic transaminase and/or glutamic oxaloacetic transaminase was defined as abnormal liver function in this study) and kidney function (An abnormal creatinine level was defined as abnormal renal function in this study) in the non-survival group was also higher than that in the survival group (*p* < 0.05).

**Table 1:** Demographic characteristics

Characteristic	Survival group	Non-survival group	*p*
Number of patients	1378	87	
Age, median (IQR), y	62.0(47.0, 72.0)	72.0(57.0, 83.0)	< 0.001
Sex, No. (%)			< 0.05
Male	756(54.9%)	61(70.1%)	
Female	622(45.1%)	26(29.9%)	
Admission type, n (%)			< 0.001
Urgent	57(4.1%)	31(35.6%)	
Emergency	139(10.1%)	28(32.2%)	
Elective	1182(85.8%)	28(32.25)	
Co-morbidities, n (%)			0.087
Hypertension	925(67.1%)	63(72.4%)	
Diabetes	633(45.9%)	38(43.7%)	
Coronary artery heart disease	205(14.9%)	15(17.2%)	
Cerebral infarction	256(18.6%)	13(15.0%)	
APACHEII score, median (IQR)	13.0(11.0, 14.0)	18.0(15.0, 22.0)	< 0.001
Mechanical ventilation, n (%)	1163(84.4%)	64(73.6%)	< 0.05
Analgesia/sedation, n (%)	1265(91.8%)	80(92.0%)	0.955
ICU length of stay, median (IQR), d	2.0(1.0, 4.0)	6.0(3.0, 14.0)	< 0.001
hospital length of stay, median (IQR), d	15.0(10.0, 23.0)	16.0(7.0, 29.0)	0.005
Discharged place (%)			< 0.001
ward	1324(96.1%)	29(33.3%)	
ICU	54(3.9%)	58(66.7%)	
Lactate, median (IQR), mmol/L			
baseline	2.5(2.0, 3.5)	4.6(2.5, 6.4)	< 0.001
24-h	2.1(1.2, 2.6)	4.8(2.3, 9.5)	< 0.001
Lactate clearance at 24 h, median (IQR),	0.36(-0.5, 0.6)	-0.14(-1.2, 0.4)	0.002
Preoperative liver function			< 0.001
Normal (%)	1311(95.0%)	72(83.0%)	
Abnormal (%)	67(5.0%)	15(17.0%)	
Preoperative renal function			< 0.001
Normal (%)	1282(93.0%)	52(60.0%)	
Abnormal (%)	96(7.0%)	35(40.0%)	
Postoperative liver function			< 0.001
Normal (%)	1252(91.0%)	63(72.0%)	
Abnormal (%)	126(9.0%)	24(28.0%)	
Postoperative renal function			< 0.001
Normal (%)	1302(94.0%)	42(48.0%)	
Abnormal (%)	76(6.0%)	45(52.0%)	

### Multiple factors associated with patient mortality

Binary logistic regression analysis was used to analyze the factors related to hospital mortality, including the APACHE II score, length of hospital stay, length of ICU stay, lactate level > 4 mmol/L at baseline, lactate clearance at 24 h, and preoperative and postoperative liver and renal function. The results showed that longer ICU stay, a higher APACHE II score, a lactate level higher than 4 mmol/L at baseline, lower lactate clearance at 24 h and postoperative renal dysfunction were risk factors for death during the hospital stay (*p* < 0.05), as shown in Table 2.

**Table 2:** Associations among multiple indicators and mortality

Variables	OR value	95%CI	*p*
ICU length of stay (days)	1.068	(1.047, 1.090)	< 0.05
hospital length of stay (days)	0.997	(0.867, 1.031)	0.511
APACHEII score	1.223	(1.177, 1.271)	< 0.05
Lactate level > 4 mmol/L (baseline)	4.326	(2.625, 7.129)	< 0.05
Lactate clearance at 24 h	0.525	(0.412, 0.669)	< 0.05
Preoperative liver function	0.805	(0.326, 1.991)	0.639
Preoperative renal function	0.607	(0.302, 1.219)	0.160
Postoperative liver function	0.624	(0.304, 1.280)	0.198
Postoperative renal function	0.104	(0.054, 0.202)	< 0.05

### The mortality rate associated with different lactate levels at baseline

**Fig. 2:**
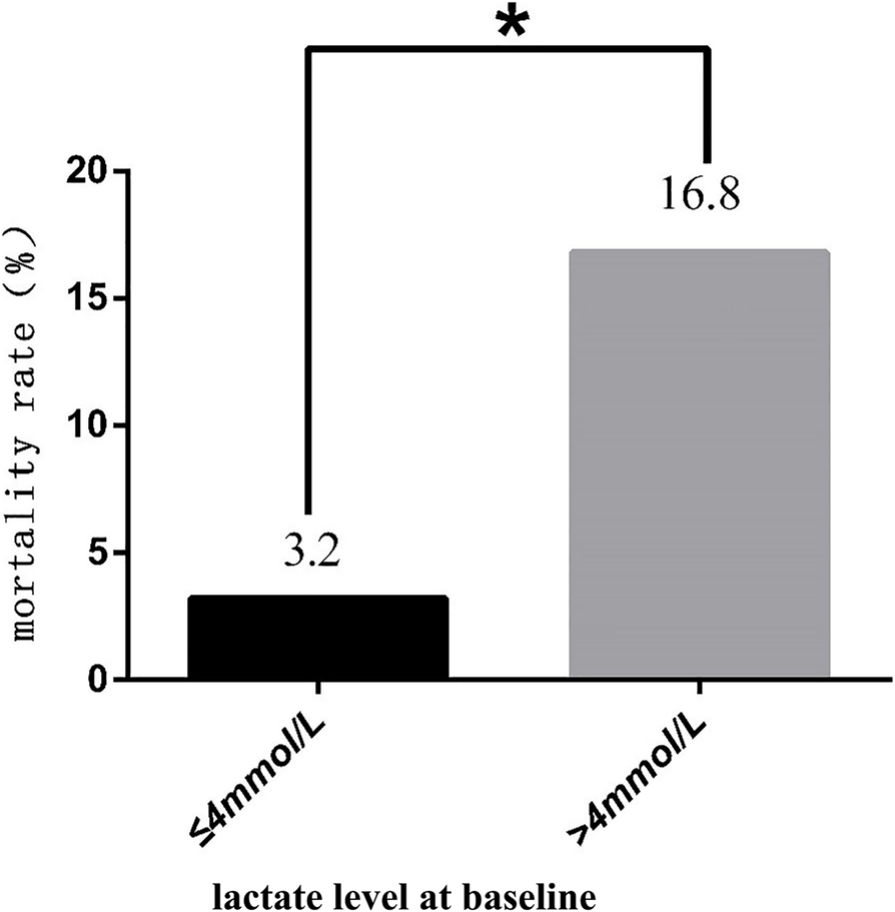
The mortality rate of patients with different lactate levels at baseline. The patients with lactate levels ≥ 4 mmol/L at baseline had a higher mortality rate. *P < 0.05 versus the group with a lactate level at baseline ≤ 4 mmol/L

Previous studies showed that a baseline lactate level > 4 mmol/L was a risk factor for death during hospitalization. In this study, the results showed that the mortality rate in the group with a baseline lactate level ≤ 4 mmol/L was significantly lower than that of the patients whose baseline lactate level was higher than 4 mmol/L (*p* < 0.05) (Fig. 2). This suggests that the higher the lactate level is at baseline, the higher the mortality rate.

### Associations between lactate clearance at 24 h and mortality

**Fig. 3:**
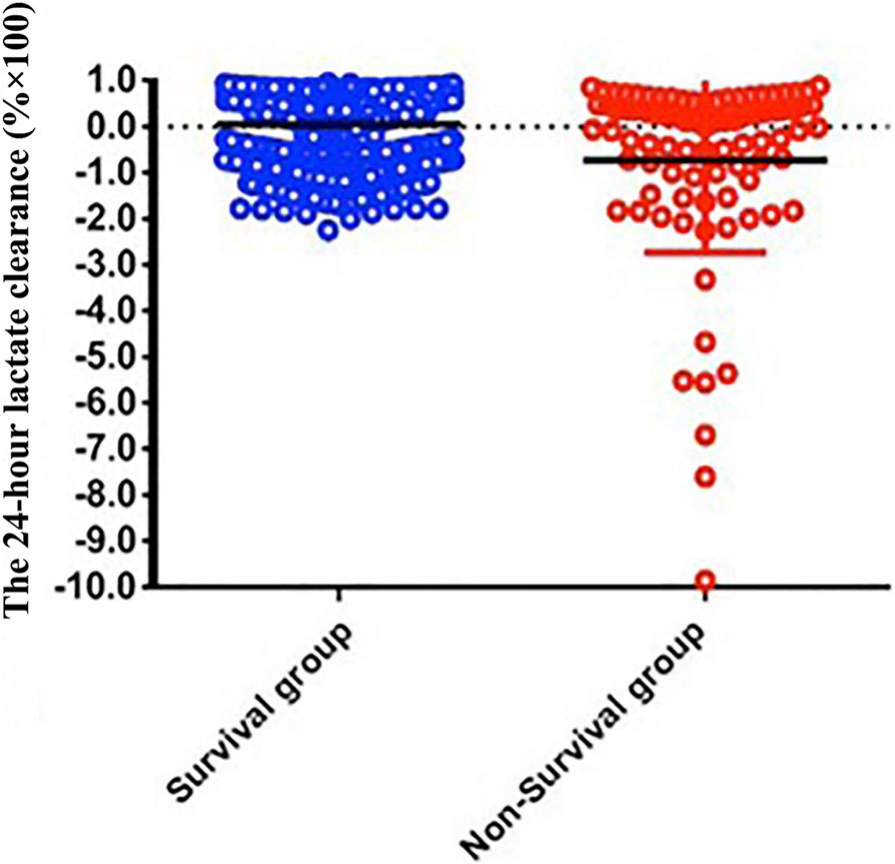
The lactate clearance at 24 h in the two groups. The lactate clearance at 24 h in the survival group was higher than that in the non-survival group

Lactate clearance at 24 h was significantly different between the survival group and the non-survival group during hospitalization. The lactate clearance at 24 h [-0.14, (-10, 1)] in the non-survival group was lower than that [0.36, (-2,1)] in the survival group (*p* < 0.05) (Fig. 3). These results suggest that the lactate clearance at 24 h was higher in the survival group and that the lower the lactate clearance was, the higher the mortality rate was.

**Fig. 4:**
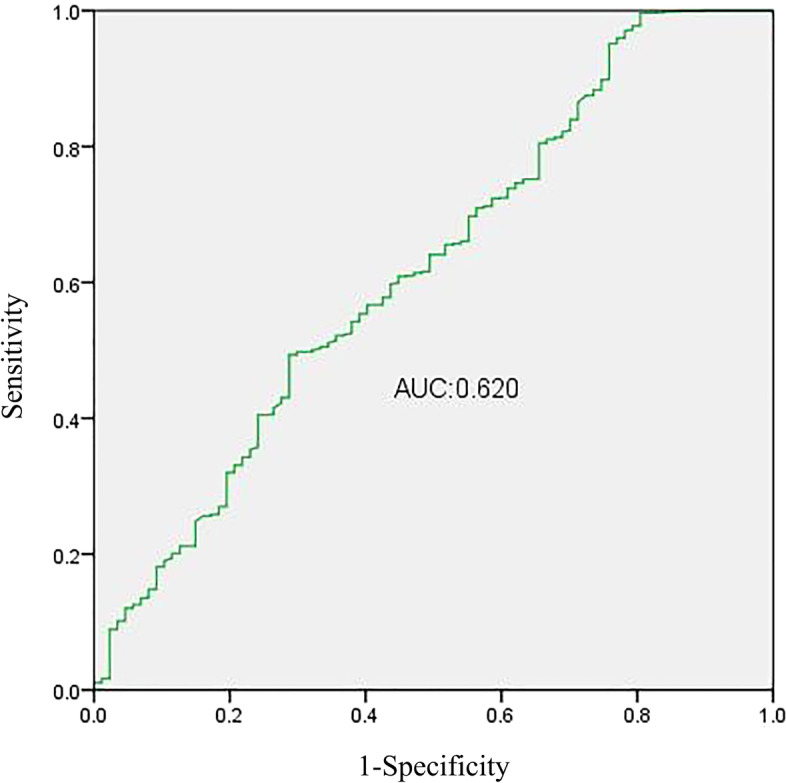
The ROC curve for lactate clearance at 24 h. The horizontal axis indicates 1- specificity, and the vertical axis indicates sensitivity. The AUC was 0.62

**Fig. 5:**
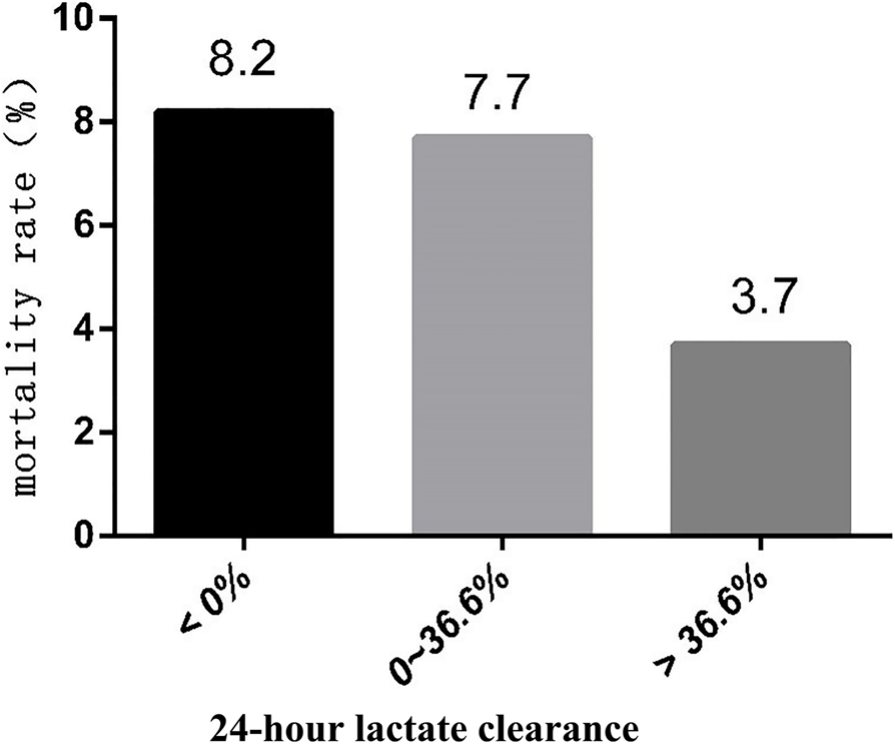
The mortality rates of patients with different lactate clearance at 24 h. Compared to the lactate clearance at 24 h < 0 group, the mortality rate of patients with a lactate clearance at 24 h > 36.6% was significantly lower. *P < 0.05 versus the group with lactate clearance at 24 h < 0

The logistic regression analysis of lactate clearance at 24 h showed that the AUC was 0.62 (95% CI: 0.556, 0.685), and the cut-off value was 0.366 (sensitivity, 0.493; specificity, 0.713; Youden’s index, 0.206) (Fig. 4). According to the cutoff value of lactate clearance at 24 h (36.6%), critically ill patients were divided into three groups. The mortality rates were 8.2, 7.7 and 3.7% in the groups with lactate clearance at 24 h < 0, 0 ~ 36.6% and > 36.6%, respectively. There were significant differences between the groups, in terms of lactate clearance at 24 h < 0 and lactate clearance at 24 h > 36.6% (*p* < 0.05). Importantly, the mortality rate was significantly lower in the group with lactate clearance at 24 h > 36.6%, while the sensitivity was low (49.3%), and the specificity was not high (71.3%) (Fig. 5).

### Associations of lactate level at baseline and APACHE II score with mortality

**Fig. 6:**
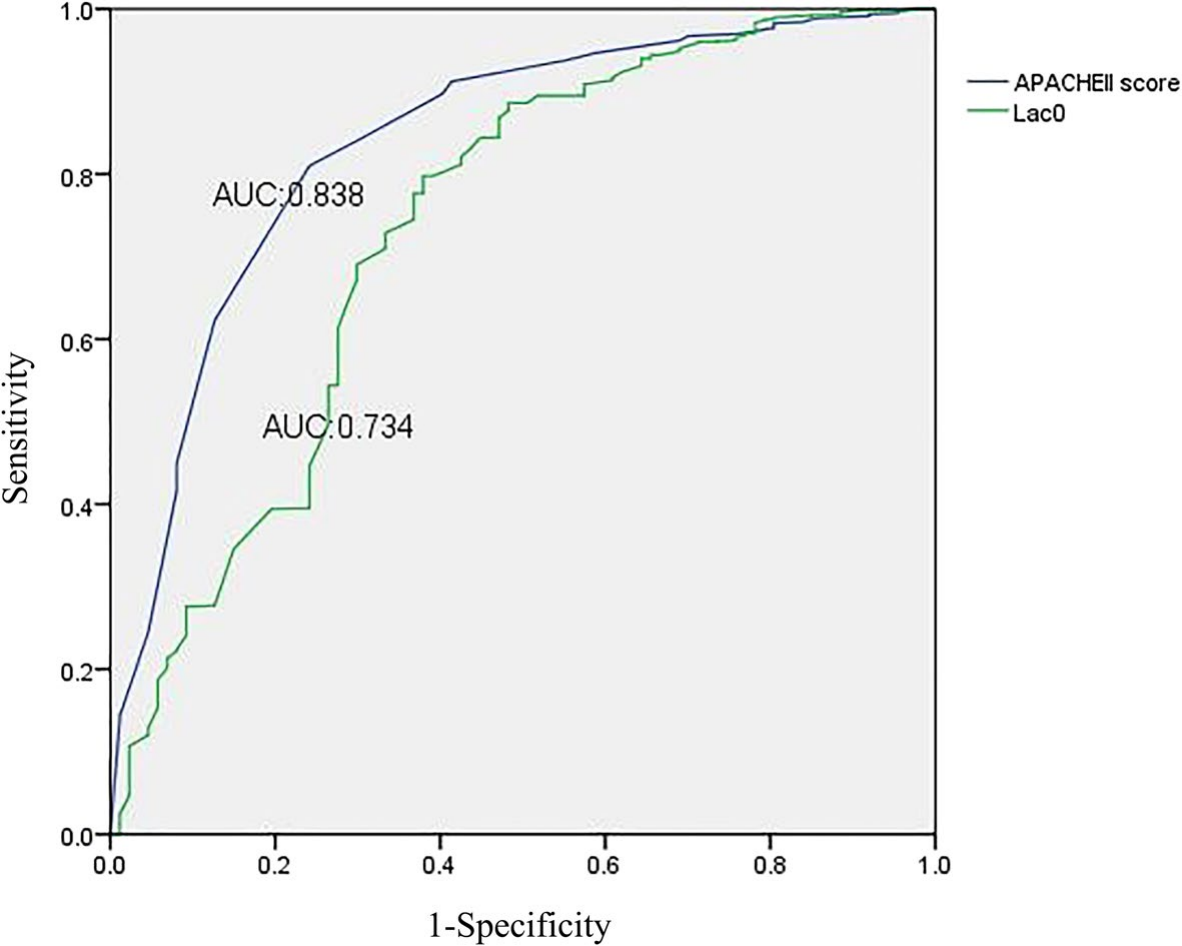
The ROC of lactate level at baseline and APACHE II score. The horizontal axis indicates 1- specificity, and the vertical axis indicates sensitivity. The blue curve represents the APACHE II score, and the AUC is 0.838. The green curve represents the lactate level at baseline, and the AUC was 0.734

The logistic regression analysis of lactate level at baseline showed that the AUC was 0.734 (95% CI: 0.671, 0.797), and the cut-off value was 3.8 (sensitivity, 0.798; specificity, 0.620; Youden’s index, 0.419, *p* < 0.001). In addition, the logistic regression analysis of the APACHE II score showed that the AUC was 0.838 (95% CI: 0.791, 0.884), and the cut-off value was 14.5 (sensitivity, 0.810; specificity, 0.759; Youden’s index, 0.569, *p* < 0.001) (Fig. 6).

### Associations of the combination of lactate level at baseline, APACHE II score, and lactate clearance at 24 h with mortality

**Fig. 7:**
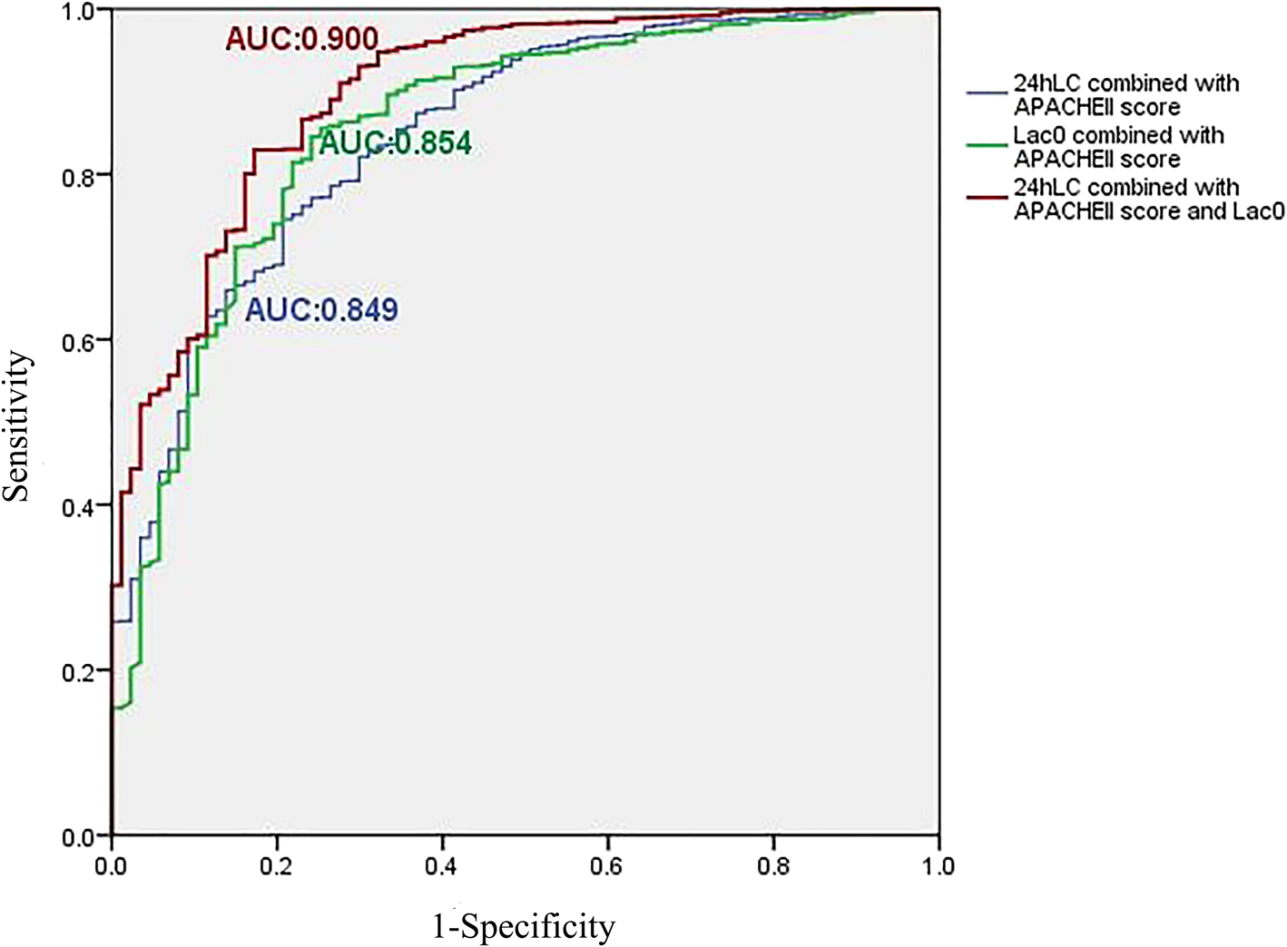
The ROC of the combination of lactate level at baseline, APACHE II score and lactate clearance at 24 h. The blue curve represents lactate clearance at 24 h combined with the APACHE II score, and the AUC is 0.849. The green curve represents the lactate level at baseline combined with the APACHE II score, and the AUC was 0.854. The red curve represents lactate clearance at 24 h combined with APACHE II score and lactate level at baseline, and the AUC is 0.854

The logistic regression analysis showed that the AUC of the ROC curve was 0.854 (95% CI: 0.810, 0.898; *p* < 0.001) for the combination of lactate level at baseline and APACHE II score. The AUC of the ROC curve was 0.849 (95% CI: 0.808, 0.890; *p* < 0.001) when the combination of lactate clearance at 24 h and APACHE II score was used. Furthermore, the AUC of the ROC curve was 0.900 (95% CI: 0.865, 0.934; *p* < 0.001) when the combination of lactate level at baseline, APACHE II score and lactate clearance at 24 h was used (Fig. 7).

### Effect of lactate level at baseline on the value of lactate level at baseline, APACHE II score, lactate clearance at 24 h for predicting mortality rate

We performed a subgroup analysis according to baseline lactate levels of > 2.1 mmol/L, > 4 mmol/L and > 10 mmol/L. The logistic regression analysis of lactate clearance at 24 h showed that the AUC increased (0.620, 0.734, 0.742, respectively) and sensitivity increased (49.3%, 82.0%, 100%, respectively), but specificity decreased significantly (71.3%, 62.0%, 58.3%, respectively). The logistic regression analysis of the lactate level at baseline showed that the AUC decreased (0.734, 0.707, 0.663, respectively), and both sensitivity (79.8%, 70.0%, 80.0%, respectively) and specificity (62.1%, 60.0%, 66.7%, respectively) were poor. In addition, the logistic regression analysis of the APACHE II score showed that the AUC decreased (0.838, 0.809, and 0.658, respectively), the sensitivity decreased (81.0, 70.0, and 50.0%, respectively), and the specificity increased significantly (75.9, 82.0, and 83.3%, respectively), as shown in Table 3.

**Table 3:** Effect of different lactate level at baseline on the predictive value for mortality rate

Variables	Lactate clearance at 24 h	Lactate level at baseline	APACHEII score
lactate level baseline(≥ 2.1 mmol/l) AUC (95%CI)	0.620(0.556, 0.685)	0.734(0.671, 0.797)	0.838(0.791, 0.884)
Cutoff value (sensitivity, specificity)	0.366(0.493, 0.713)	3.800(0.798, 0.621)	14.500(0.810, 0.759)
baseline(≥ 4 mmol/l) AUC (95%CI)	0.734(0.651, 0.816)	0.707(0.627, 0.787)	0.809(0.753, 0.865)
Cutoff value (sensitivity, specificity)	0.366(0.820, 0.620)	5.750(0.700, 0.600)	15.500(0.700, 0.820)
baseline(≥ 10mmo/l) AUC (95%CI)	0.742(0.522, 0.962)	0.663(0.470, 0.908)	0.658(0.421, 0.896)
Cutoff value (sensitivity, specificity)	0.267(1.000, 0.583)	13.400(0.800, 0.667)	16.500(0.500, 0.833)

## Discussion

From the above results, it can be concluded that the AUC of lactate level at baseline in addition to APACHE II score was higher than that of lactate clearance at 24 h, and the AUC of the APACHE II score was higher than that of the lactate level at baseline. Therefore, the APACHE II score has a superior predictive value for patients with abnormal lactate levels in the ICU. What is more important, the combination of the lactate level at baseline, APACHE II score and lactate clearance at 24 h has the highest value for predicting mortality rate in critically ill patients and is superior to the use of any of the indicators alone.

Mortality is very high in critically ill patients due to their complex condition and rapid progression; thus, it is extremely important to identify some indicators to predict prognosis. In previous studies, researchers have confirmed that there is a certain correlation between the lactate level and the mortality rate of critically ill patients [31]. In fact, lactate levels may be more closely related than hemodynamic changes to the prognosis of patients in the early stage of resuscitation [32]. Lactate levels can be widely used to guide the resuscitation of critically ill patients [27]. It has been confirmed in previous studies that a decrease in lactate levels was consistent with a better prognosis, regardless of whether the patient had sepsis [33]. Lactate clearance is also widely used to predict the prognosis of critically ill patients, but its predictive value is still controversial [34, 35].

In this retrospective cohort study, we investigated the association between lactate levels and mortality rates in critically ill patients. The results indicated that the mortality rate of patients increased significantly when lactate levels were higher than normal and lactate clearance at 24 h was low. However, considering that lactate level and lactate clearance are closely related to liver and renal function, we analyzed some other indicators, such as the APACHE II score. We found that the lactate level at baseline, the APACHE II score and the lactate clearance at 24 h were all important factors related to patient prognosis. In the non-survival group, the proportions of elderly and male patients were higher, but, when age and gender were included in analyzing the association of the parameters (lactate, lactate clearance, and APACHE-II score) with survival the analysis parameters, the sensitivity and specificity were low. In addition, our results showed that the AUC of model (age, sex APACHE-II score, and lactate clearance) was not significantly higher than that of model (age, sex, and APACHE-II score). Lactic acidosis occurs when lactate level > 4 mmol/l. The mortality rate was significantly higher when the lactate level at baseline was higher than 4 mmol/L. Compared with the survival group, the non-survival group had a higher baseline lactate level and APACHE II score and longer ICU and hospital stays. In addition, lactate clearance at 24 h was lower in the non-survival group than in the survival group.

However, the lactate value at baseline, APACHE II score and lactate clearance at 24 hrs for predicting the prognosis of critically ill patients is still not very clear. Therefore, we analyzed the associations of the lactate level at baseline, APACHE II score, and lactate clearance at 24 hrs with the mortality rate. The results showed that the predictive value of lactate clearance at 24 hrs was not high, and its sensitivity and specificity were lower. The predictive value was not high for lactate clearance at 24 hrs, lactate level at baseline and APACHE II score alone in predicting the prognosis of patients in the ICU and that there is no statistically significant difference in mortality in the subgroup with a baseline lactate level >10 mmol/L. The predictive value of the baseline lactate level plus the APACHE II score was higher than that of the lactate clearance at 24 hrs, and the APACHE II score was more valuable than the lactate level at baseline for predicting the prognosis of critically ill patients with abnormal lactate levels. A clinical trial showed that the mortality rate increased as the APACHE II score increased, and the APACHE II score had a better value for predicting patient prognosis [36]. In this study, the results showed that although the AUC of the APACHE II score for predicting prognosis was higher (0.838), its sensitivity and specificity were somewhat poor (81.0 and 75.9%, respectively). Further analysis showed that the predictive value of the lactate level at baseline combined with the APACHE II score was higher than that of the lactate level at baseline or the APACHE II score alone. In addition, the predictive value of lactate clearance at 24 hrs combined with the APACHE II score was also significantly higher than that of lactate clearance at 24 hrs or the APACHE II score alone. In particular, the AUC reached 0.900, the predictive value was markedly higher than that of any of these indictors alone, and the sensitivity and specificity were better when these three indicators were combined. Therefore, the combination of lactate level at baseline, lactate clearance and the APACHE II score best predicted the prognosis of critically ill patients in the early stage.

There are still some limitations in this study. First, the number of cases included in this study was limited, and the sample size was not sufficient. All patients included in the study had abnormal lactate levels and ICU stays longer than 24 h. Thus, whether the related indicators can be used for prognosis in other departments, such as the emergency department, is still unknown. Second, we analyzed the mortality rate of critically ill patients during hospitalization and did not follow up to determine the outcomes of patients discharged at the later stage; consequently, it was impossible to perform a survival analysis. In addition, we did not analyze the causes of death in the study. Third, this study was a retrospective, non-double-blind trial, and bias and unknown confounding factors were inevitable. Fourth, a dynamic assessment of APACHE II scores was not performed in this study. Fifth, we only analyzed lactate levels at baseline and at 24 h, and further analysis of lactate levels more than twice within 24 h should be conducted. Therefore, a large sample size and multicenter, double-blind, randomized trials are needed to provide more valuable, evidence-based indicators for predicting prognosis.

## Conclusions

Critically ill patients have a high mortality due to their complex condition and rapid disease progression. This was a retrospective, cohort study and to identify some indicators to predict the prognosis. We found that the combination of lactate level, lactate clearance and the APACHE II score better predicts short-term outcomes in critically ill patients, which is beneficial to the management of critical patients.

## Data Availability

The datasets used and/or analyzed during the current study are available from the corresponding author on reasonable request.

## Abbreviations

SIRS: Systemic inflammatory response syndrome
ICU: Intensive care unit
APACHE: Acute Physiology and Chronic Health Evaluation
LC: Lactate clearance
ROC: Receiver operating characteristic
AUC: Area under curve
